# Vitamin K and vascular calcification: from biological plausibility to clinical realism

**DOI:** 10.1093/ckj/sfag249

**Published:** 2026-08-05

**Authors:** Vincent Brandenburg, Nikolaus Keil, Thilo Krüger, Hermann Salmhofer, Turgay Saritas

**Affiliations:** Department of Cardiology and Nephrology, Rhein-Maas Klinikum, Würselen, Germany; Medical University of Vienna, Center for Public Health, Department of Epidemiology, Vienna, Austria; DaVita Clinical Research Germany, Duesseldorf, Germany; Department of Nephrology and Clinical Immunology, University Hospital RWTH Aachen, Aachen, Germany; Department of Internal Medicine I, Paracelsus Medical University, Salzburg, Austria; Department of Nephrology and Clinical Immunology, University Hospital RWTH Aachen, Aachen, Germany; Department of Internal Medicine, Nephrology, and Transplantation, Erasmus Medical Center, Rotterdam, The Netherlands

**Keywords:** chronic kidney disease, coronary artery calcification, matrix Gla protein, vascular calcification, vitamin K

## Abstract

Vitamin K has long been considered a potentially protective factor against ectopic calcification. This review summarizes the biological rationale and randomized clinical evidence for vitamin K supplementation as an anti-calcification strategy, with particular emphasis on chronic kidney disease. The concept is biologically plausible because vitamin K is required for post-translational γ-carboxylation and activation of matrix Gla protein, a key endogenous inhibitor of vascular and valvular calcification. Patients with chronic kidney disease frequently exhibit functional vitamin K deficiency and accelerated vascular and valvular calcification, providing a strong rationale for intervention trials. Randomized studies have therefore examined whether vitamin K1 or vitamin K2, mainly menaquinone-7, can slow progression of coronary, aortic, or valvular calcification. Overall, the evidence remains mixed. Both vitamin K1 and K2 supplementation consistently improves biochemical markers of vitamin K status. However, this biochemical response has not translated uniformly into attenuation of calcification progression on imaging. Across available randomized trials, vitamin K1 has generated the more consistent, although still heterogeneous, evidence for slowing imaging-based calcification progression, particularly in selected high-risk cohorts and in haemodialysis populations receiving intensive dosing. By contrast, vitamin K2 has not produced reproducible benefits on CT-based vascular or valvular calcification endpoints. Importantly, neither vitamin K1 nor vitamin K2 has demonstrated reductions in major adverse cardiovascular events or mortality. Where clinical events were collected, studies were underpowered and results were neutral. At present, vitamin K supplementation remains pharmacodynamically active but clinically unproven as a strategy to prevent cardiovascular endpoints in chronic kidney disease.

## INTRODUCTION

Vitamin K is best known for its role in haemostasis. Most clinicians encounter vitamin K through its function as an essential cofactor for γ-carboxylation of coagulation factors and through the clinical use of vitamin K antagonists. Its role in cardiovascular biology was recognized later, largely through the discovery that matrix Gla protein (MGP) is a vitamin K-dependent inhibitor of ectopic calcification (Fig. 1). A landmark experimental study showed that mice lacking MGP developed severe arterial and cartilage calcification and died early from vascular complications [1]. These findings established MGP as a local inhibitor of mineral deposition in tissues that must remain elastic, particularly the arterial wall and cartilage. MGP is synthesized by vascular smooth muscle cells and chondrocytes and requires post-translational activation. Similar to coagulation factors II, VII, IX and X, this activation depends on vitamin K-dependent γ-carboxylation.

**Figure 1:**
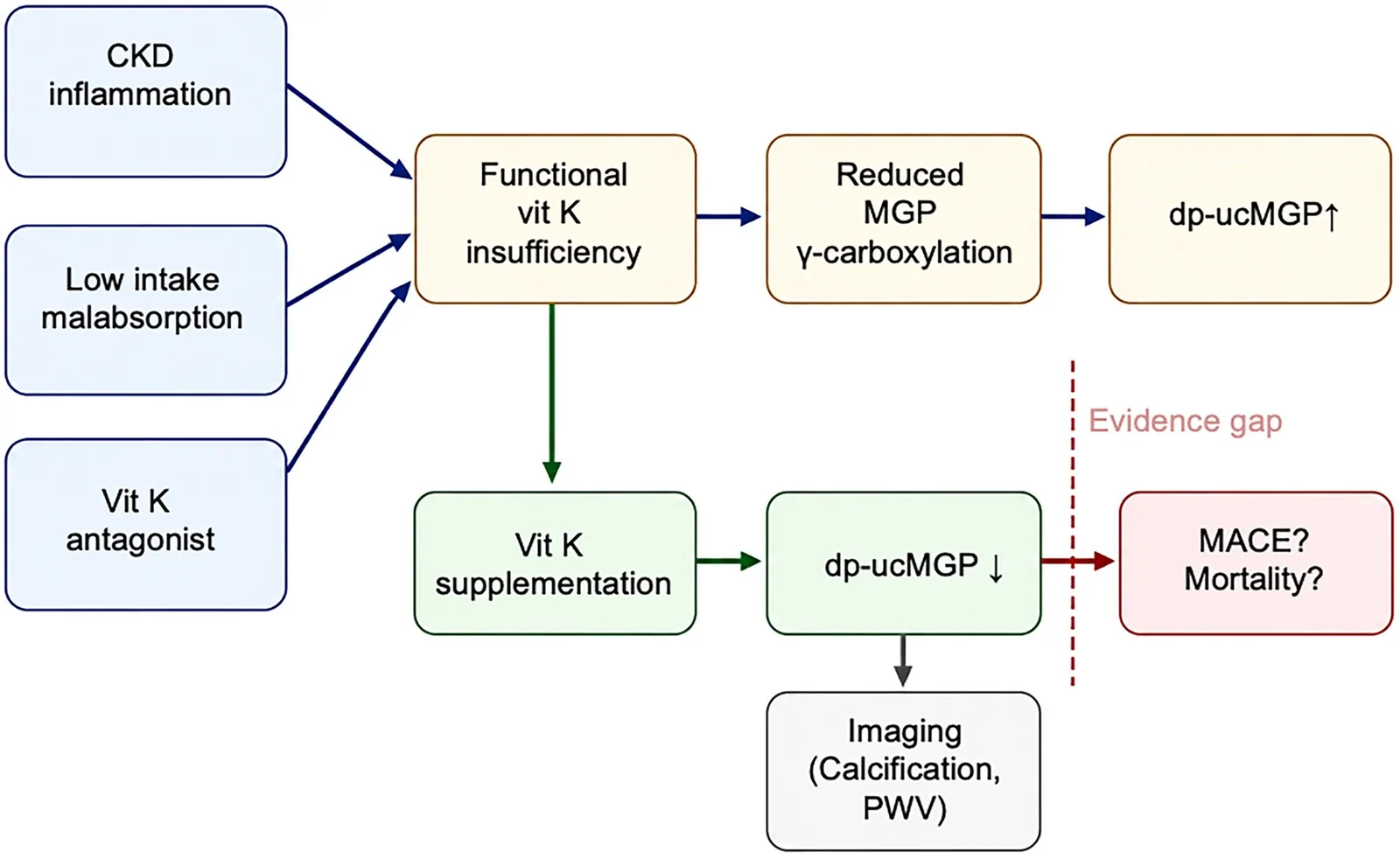
Vitamin K, matrix Gla protein and the translational evidence gap. Vitamin K is required for γ-carboxylation and activation of matrix Gla protein, an endogenous inhibitor of ectopic mineralization. Chronic kidney disease, dialysis, poor intake, impaired absorption and vitamin K antagonist exposure may contribute to functional vitamin K deficiency, reflected by elevated dp-ucMGP. Although vitamin K supplementation consistently lowers dp-ucMGP, randomized trials have not established a reproducible effect on CT-based calcification progression or cardiovascular outcomes. The key evidence gap is therefore the translation from biomarker correction to clinical benefit. Abbreviations: CKD, chronic kidney disease; dp-ucMGP, dephosphorylated-uncarboxylated matrix Gla protein; MACE, major adverse cardiovascular events; MGP, matrix Gla protein; PWV, pulse wave velocity.

Human genetic observations further support the biological relevance of MGP. Loss-of-function mutations in the MGP gene cause Keutel syndrome, a rare disorder characterized by abnormal cartilage calcification and vascular abnormalities [2]. Together, experimental and human genetic data provide a compelling biological rationale: if inactive MGP promotes ectopic calcification, then improving vitamin K availability might enhance MGP activity and reduce vascular calcification.

Patients with chronic kidney disease (CKD) represent an especially relevant target population. They frequently exhibit accelerated cardiovascular calcification, including both intimal calcification related to atherosclerosis and medial calcification related to arterial stiffness. They also often show biochemical evidence of functional vitamin K deficiency. Approximately a decade ago, this combination of biological plausibility and clinical need generated considerable enthusiasm for vitamin K supplementation as a potential anti-calcification strategy [3]. Since then, several prospective randomized trials have been completed.

The central question is therefore no longer whether vitamin K biology is plausible. The question is whether vitamin K supplementation meaningfully alters vascular calcification progression or cardiovascular outcomes in patients at risk, particularly those with CKD. This review summarizes the current interventional evidence and discusses why initial enthusiasm should now be balanced by clinical realism.

## IS THERE A WELL-DEFINED STATE OF VITAMIN K DEFICIENCY?

Clinically manifest vitamin K deficiency is uncommon in developed countries, except in settings such as malnutrition, malabsorption, prolonged antibiotic exposure, cholestatic liver disease, or advanced age. However, the concept of subclinical or functional vitamin K deficiency has gained attention in relation to bone and vascular health [4, 5].

In bone, the relevant vitamin K-dependent protein is osteocalcin. Osteocalcin is produced by osteoblasts and becomes fully functional after γ-carboxylation. In the vasculature, the central vitamin K-dependent protein is MGP. Osteocalcin and MGP are therefore related but not identical. Both are vitamin K-dependent Gla proteins, but osteocalcin primarily reflects bone metabolism, whereas MGP is a key inhibitor of vascular and valvular calcification. A potential synergistic interaction between vitamin K and vitamin D on skeletal outcomes has been suggested, including improvements in bone mineral density and reductions in undercarboxylated osteocalcin, particularly with vitamin K2 supplementation [6].

In contrast to vitamin D, where serum 25-hydroxyvitamin D provides an established marker of vitamin D status, no universally accepted biomarker or threshold defines vitamin K sufficiency [7]. Several markers have been used, including circulating phylloquinone, menaquinones, protein induced by vitamin K absence or antagonist-II, undercarboxylated osteocalcin and dephosphorylated-uncarboxylated MGP. Among these, dephosphorylated-uncarboxylated MGP (dp-ucMGP) has become the most widely used vascular marker [8, 9].

If MGP remains uncarboxylated, this suggests insufficient vitamin K availability for its activation. Elevated dp-ucMGP is therefore generally interpreted as a marker of functional vitamin K deficiency, particularly at the vascular level. Most vitamin K intervention trials have shown that supplementation lowers dp-ucMGP, confirming biological uptake and pharmacodynamic activity.

However, this should not be confused with validation as a therapeutic target. No consensus exists regarding insufficient, optimal or harmful dp-ucMGP levels. It is also unclear how far dp-ucMGP should be lowered and whether a given reduction in dp-ucMGP predicts less calcification progression or fewer cardiovascular events. At present, dp-ucMGP should therefore be regarded as a useful pharmacodynamic marker of vitamin K activity, but not as a validated surrogate endpoint for cardiovascular benefit.

## WHAT IS THE OPTIMAL APPROACH TO VITAMIN K SUPPLEMENTATION?

Vitamin K is not a single compound. It refers to a group of structurally related molecules that share a 2-methyl-1,4-naphthoquinone core structure but differ in their side chains, pharmacokinetics, and tissue distribution [5]. Vitamin K1, or phylloquinone, is mainly obtained from green leafy vegetables. Vitamin K2 refers to menaquinones, including MK-4 to MK-13, which are found in fermented foods and animal products and are also produced by bacteria. Among menaquinones, MK-7 is the most frequently used compound in clinical supplementation studies. Enzymatic conversion of K1 to K2, particularly MK-7, appears to occur only to a limited extent in humans. Of note, the all-trans configuration of MK-7 exhibits markedly higher carboxylative efficacy of osteocalcin and MGP than *cis* isomers [10].

To date, no dietary intervention trial has systematically compared a vitamin K-rich diet (e.g. increased intake of green vegetables) with pharmacological supplementation, nor is it known how these two strategies might differentially affect dp-ucMGP levels. In human interventional studies, both K1 and K2 have been administered. At present, it remains uncertain whether one form is clinically superior to the other in terms of efficacy. In a comparative analysis, Andrian *et al*. [9] reported that K2 supplementation resulted in a greater increase in circulating vitamin K levels, whereas K1 appeared more effective in lowering ucMGP. The latter observation is most likely dose dependent. A small randomized trial directly comparing vitamin K1, vitamin K2 and placebo in haemodialysis patients focused on MGP-related biochemical markers rather than imaging or clinical endpoints [11]. Thus, direct comparative evidence remains insufficient. To our knowledge, no clinical trial has yet investigated potential differences between K1 and K2 with respect to vascular calcification or other clinically meaningful endpoints beyond biochemical markers.

A further practical issue is regulatory. Depending on jurisdiction, vitamin K1 is available as a medicinal product, whereas MK-7 is often marketed as a dietary supplement. This may influence trial design, quality control, dosing reliability, and regulatory feasibility. Consequently, good clinical practice regulations, for example in Germany, require investigators to employ K1 rather than K2 in interventional studies.

## IS VITAMIN K SUPPLEMENTATION SAFE?

Available human intervention trials have not raised major safety concerns regarding vitamin K supplementation. This is clinically important, as vitamin K is fat-soluble and might theoretically accumulate. In practice, vitamin K appears to have a relatively favourable safety profile. Several trials investigating the use of vitamin K in osteoporosis employed high doses that far exceeded the recommended daily allowance [12]. Even under these conditions, no increase in thromboembolic risk was observed, although the studies also failed to demonstrate a robust reduction in osteoporotic fractures. The hepatic coagulation system is likely already saturated under physiological conditions, and additional vitamin K does not appear to further increase coagulation activity in individuals not treated with vitamin K antagonists. Unlike vitamin D, vitamin K does not appear to undergo substantial storage in adipose tissue, which likely contributes to its relatively short half-life. Nevertheless, safety statements should be made with caution. Vitamin K supplementation can interfere with vitamin K antagonist therapy and may destabilize anticoagulation in patients receiving warfarin or phenprocoumon.

## DOES VITAMIN K ANTAGONIST TREATMENT PROMOTE VASCULAR CALCIFICATION?

If vitamin K supplementation were to exert anti-calcification effects, the opposite might be expected for treatment with vitamin K antagonists. Observational studies supported this hypothesis, suggesting a potential causal role of vitamin K antagonists in the development of tracheal and vascular calcification [13–15]. Moreover, vitamin K antagonist use has been implicated in the pathogenesis of calciphylaxis, a devastating disorder characterized by calcification of small cutaneous arteries [15].

However, randomized data have been less straightforward. Trials comparing vitamin K antagonists with direct oral anticoagulants have not consistently shown attenuation of calcification progression after avoiding vitamin K antagonism. In the IRIVASC trial, 192 patients requiring long-term oral anticoagulation were randomized to phenprocoumon or rivaroxaban, with serial CT assessment of coronary calcification. Coronary calcification progressed over time in both groups, without a clear difference between strategies [16].

While IRIVASC enrolled patients without CKD, the VALKYRIE trial [17] addressed a similar question in haemodialysis patients. Patients were randomized to vitamin K antagonist therapy, rivaroxaban or rivaroxaban plus high-dose MK-7. Avoidance of vitamin K antagonism combined with vitamin K2 supplementation might, in theory, have been expected to produce the strongest anti-calcification signal. However, over 18 months, progression of coronary artery, thoracic aortic and cardiac valve calcification, as well as pulse wave velocity, did not differ significantly between groups. Likewise, clinical outcomes, including all-cause mortality, stroke, and cardiovascular event rates, were similar between arms. Bleeding events did not differ overall, except for a lower incidence of life-threatening and major bleeding in the rivaroxaban groups compared with VKA. The authors concluded that discontinuation of VKAs and addition of high-dose vitamin K2 provided no significant benefit on vascular calcification progression.

These trials do not prove that vitamin K antagonists are irrelevant to vascular calcification. Rather, they show that replacing vitamin K antagonists with direct oral anticoagulants, with or without vitamin K2 supplementation, is not sufficient to reliably slow established calcification over the studied time frames. Established calcification in CKD and dialysis may be too advanced, too multifactorial or too slowly modifiable for short-term anticoagulation changes to translate into detectable imaging benefits.

## IS VASCULAR CALCIFICATION A RELIABLE SURROGATE FOR CARDIOVASCULAR HEALTH?

There is no doubt that vascular calcification is associated with cardiovascular risk. Coronary artery calcium predicts cardiovascular events in the general population, and calcification progression is associated with incident coronary heart disease [18]. In CKD, vascular calcification is particularly common and contributes to arterial stiffness, increased pulse wave velocity, left ventricular hypertrophy, and cardiovascular mortality. Nevertheless, vascular calcification is not a simple linear surrogate for cardiovascular benefit. Calcification can reflect advanced disease burden, but it may also contribute to plaque stabilization in certain settings. Calcified plaques are generally less prone to rupture than lipid-rich, inflamed, non-calcified plaques. Thus, the biological meaning of calcification depends on plaque type, location, density, distribution, and disease stage [19].

The experience with statin therapy illustrates this paradox. Statins reduce cardiovascular events, yet they have little effect on, and in some analyses may even increase, CT-based coronary calcium progression [20, 21]. This does not imply that statins are harmful. Rather, it suggests that increasing calcification density may sometimes accompany plaque stabilization. Therefore, a therapy that does not reduce Agatston score progression may still be clinically beneficial, while a therapy that reduces calcification progression may not necessarily reduce events.

This distinction is central for interpreting vitamin K trials. Most studies have relied on CT-based calcification scores, arterial stiffness, or dp-ucMGP rather than hard clinical endpoints. These are useful mechanistic and intermediate measures, but they cannot establish cardiovascular benefit. Moreover, current imaging rarely distinguishes sufficiently between medial calcification, intimal calcification, calcified plaque volume, calcification density and active microcalcification. Future studies should consider advanced imaging approaches, including plaque composition, calcification density, and 18F-NaF PET activity, rather than relying only on total calcium burden.

## HUMAN INTERVENTION TRIALS: WHAT HAVE WE LEARNED?

In recent years, several vitamin K interventional trials have been published with largely comparable designs (Table 1). These were randomized, prospective studies that used computed tomography to assess cardiovascular calcification (aortic valve and/or CAC) with follow-up durations of 12–24 months. Most trials demonstrated longitudinal reductions in dp-ucMGP levels with vitamin K supplementation. However, results varied according to the vitamin K form and dosage, as well as the patient populations studied, including healthy, non-dialyse CKD, and dialysis cohorts.

**Table 1:** Randomized vitamin K intervention trials with vascular calcification, calcification propensity, or vascular stiffness endpoints.

Study	Population	Intervention	Duration	Main vascular endpoint	Main result	Interpretation
Shea *et al*.	Older adults, non-CKD	Vitamin K1, 500 µg/day	3 years	CAC progression	No significant effect in the overall ITT analysis; lower CAC progression in adherent participants and in those with baseline CAC >10	Supportive but subgroup-/adherence-dependent
Bellinge *et al*./ViKCoVaC post hoc	Diabetes mellitus, CAC >10, no clinical CAD	Vitamin K1, 10 mg/day	3 months	New 18F-NaF PET-positive coronary/aortic lesions	Lower odds of developing new PET-positive lesions	Suggests effect on active microcalcification; hypothesis-generating
Diederichsen *et al*./AVADEC	Elderly men with AVC >300 AU	MK-7, 720 µg/day + vitamin D	24 months	AVC progression	No reduction in AVC progression	Neutral primary endpoint
Hasific *et al*./AVADEC secondary analysis	Secondary CAC analysis of AVADEC	MK-7, 720 µg/day + vitamin D	24 months	CAC progression/plaque volume	Overall neutral; signal in participants with high baseline CAC	Hypothesis-generating subgroup signal
Zwakenberg/Bartstra *et al*.	Type 2 diabetes with CVD	MK-7, 360 µg/day	6 months	18F-NaF PET activity and CT-based systemic arterial calcification	No significant effect on arterial calcification progression	Neutral
Kurnatowska *et al*.	Non-dialysis CKD stages 3–5	MK-7 + vitamin D	270 days	CIMT/calcification-related measures	Possible effect on atherosclerosis progression; no clear calcification benefit	Small, hypothesis-generating
Witham *et al*./K4Kidneys	CKD stages 3b-4	MK-7, 400 µg/day	12 months	PWV/vascular stiffness; AAC	No improvement in vascular stiffness or other vascular health measures	Neutral; not primarily a calcification-imaging trial
Lees *et al*./ViKTORIES	Kidney transplant recipients	Menadiol diphosphate, 5 mg three times weekly	12 months	Aortic distensibility/CAC	No reduction in vascular stiffness or calcification	Neutral
Eelderink *et al*.	Vitamin K-deficient kidney transplant recipients	MK-7, 360 µg/day	12 weeks	T50/PWV	No effect on T50; reduced PWV progression	Functional vascular signal; no calcification-imaging endpoint
Holden *et al*./iPACK-HD	Haemodialysis, CAC ≥ 30 AU	Vitamin K1, 10 mg three times weekly	12 months	Feasibility; CAC progression	Improved vitamin K status; no difference in CAC progression	Feasibility/pilot trial; neutral for CAC
Saritas *et al*./VitaVasK	Haemodialysis with established CAC	Vitamin K1, 5 mg three times weekly	18 months	CAC/TAC progression	Marked attenuation of aortic calcification progression; CAC signal favourable	Positive imaging signal, but small, open-label, early stopped
Macias-Cervantes *et al*.	Haemodialysis, CAC > 10 AU	Intravenous vitamin K1, 10 mg three times weekly	12 months	CAC progression	Lower CAC progression versus placebo; RR CI crossed 1	Supportive K1 signal; needs confirmation
Oikonomaki *et al*.	Haemodialysis	MK-7, 200 µg/day	12 months	Abdominal aortic calcification	Lower ucMGP; no significant calcification benefit	Neutral; high dropout/open-label
Levy-Schousboe *et al*./RenaKvit	Dialysis	MK-7, 360 µg/day	24 months	PWV, CAC, AAC	Improved vitamin K status; no reduction in arterial calcification progression	Neutral
Haroon *et al*./Trevasc-HDK	Haemodialysis	MK-7, 360 µg three times weekly	18 months	CAC progression/vascular calcification	Lower dp-ucMGP; no benefit on vascular calcification, MACE or mortality	Neutral
Naiyarakseree *et al*.	Haemodialysis	MK-7, 375 µg/day	24 weeks	Arterial stiffness	No overall PWV benefit; possible diabetes subgroup signal	Not a calcification endpoint; optional row

Abbreviations: AAC, abdominal aortic calcification; AU, Agatston units; AVC, aortic valve calcification; CAC, coronary artery calcification; CAD, coronary artery disease; CKD, chronic kidney disease; CIMT, carotid intima-media thickness; CT, computed tomography; CVD, cardiovascular disease; dp-ucMGP, dephosphorylated-uncarboxylated matrix Gla protein; HD, haemodialysis; ITT, intention-to-treat; MACE, major adverse cardiovascular events; MK-7, menaquinone-7; PET, positron emission tomography; PWV, pulse wave velocity; RR CI, relative risk confidence interval; TAC, thoracic aortic calcification; 18F-NaF, fluorine-18 sodium fluoride.

### General and high-risk non-CKD populations

In non-CKD populations, vitamin K1 has shown some favourable signals. Shea and colleagues randomized older adults to vitamin K1 supplementation and found attenuation of coronary artery calcification progression among participants with pre-existing calcification at baseline [22]. A more dynamic imaging approach was used by Bellinge and colleagues in individuals with diabetes mellitus. In a post hoc analysis of a randomized trial, vitamin K1 supplementation reduced the development of new 18F-NaF PET-positive lesions in the coronary arteries and aorta [23]. Since 18F-NaF PET detects active microcalcification, this finding is conceptually important. It suggests that vitamin K may influence early calcification activity even when changes in established CT calcium scores are difficult to detect over short follow-up. In a randomized placebo-controlled trial of postmenopausal women, the regimen containing vitamin K1 in addition to vitamin D and minerals showed the most favourable effect, attenuating the deterioration in carotid arterial elasticity over three years compared with placebo. However, no significant effect was observed on carotid intima–media thickness, and vitamin D plus minerals without vitamin K1 did not differ from placebo [24].

For vitamin K2, results have been less encouraging. In the AVADEC trial, elderly men with aortic valve calcification were randomized to MK-7 plus vitamin D vs placebo. After 2 years, supplementation did not reduce progression of aortic valve calcification [23]. A secondary analysis of this trial also found no significant reduction in overall coronary artery calcium progression, although a subgroup with high baseline coronary calcium ≥400 AU showed a benefit [25]. Similarly, in patients with type 2 diabetes and cardiovascular disease, MK-7 did not halt progression of systemic arterial calcification [26]. In a double-blind randomized trial of 80 older adults with established vascular disease, 6 months of MK-7 increased circulating vitamin K and significantly reduced dp-ucMGP compared with placebo. However, supplementation did not improve endothelial function, arterial stiffness, other vascular measures, or physical performance [27]. A recently reported conference abstract suggested that MK-7 might slow coronary calcification progression in hypertensive patients with mild-to-moderate coronary artery disease [28, 29]. However, as this evidence is currently available only in abstract form, with limited methodological detail, it should not drive clinical conclusions.

### Non-dialysis CKD and kidney transplantation

In non-dialysis CKD, interventional data remain limited and heterogeneous. Kurnatowska and colleagues studied MK-7 in patients with CKD stages 3–5 and reported potential effects on atherosclerosis progression, but no clear evidence of reduced vascular calcification [30]. The K4Kidneys trial tested MK-7 in patients with CKD stages 3b-4 and found no improvement in vascular stiffness or other measures of vascular health over 1 year [31].

Kidney transplant recipients represent another relevant population, as vascular calcification and arterial stiffness remain common after transplantation. The ViKTORIES trial found that menadiol diphosphate did not reduce vascular stiffness or calcification over 12 months in a heterogeneous cohort of prevalent kidney transplant recipients [32]. Because menadiol is a synthetic precursor requiring conversion to MK-4, these neutral findings should be extrapolated cautiously to K1 or MK-7. A trial with MK-7 in vitamin K-deficient kidney transplant recipients did not improve serum calcification propensity over 12 weeks, although progression of arterial stiffness was attenuated [33]. These findings suggest that selection based on vitamin K status may be biologically reasonable, but the clinical relevance remains uncertain.

### Dialysis populations

Dialysis patients have the highest burden of vascular calcification and the most pronounced biochemical evidence of functional vitamin K deficiency. They are therefore an attractive population for intervention trials. However, they also have advanced, multifactorial calcification driven by disordered mineral metabolism, inflammation, oxidative stress, uraemic toxins, dialysis vintage, diabetes, and vascular injury. This may make calcification difficult to modify.

Early randomized studies with vitamin K2 in haemodialysis established that inactive vitamin K-dependent proteins can be lowered by supplementation [34, 35]. These studies were important pharmacodynamic proof-of-concept studies, but they were not designed to prove attenuation of vascular calcification.

Several trials have investigated vitamin K1 in haemodialysis. The iPACK-HD study was a randomized, placebo-controlled pilot trial of vitamin K1 in patients with end-stage kidney disease and baseline coronary calcification [36]. Vitamin K1 improved vitamin K status, but did not significantly reduce coronary artery calcification progression over 12 months. The study demonstrated feasibility but was not powered to establish clinical benefit.

Macias-Cervantes *et al*. [37] studied 60 haemodialysis patients with CAC >10 AU, randomized to K1 (10 mg three times per week) or placebo for 12 months. Median baseline CAC scores were comparable between groups. After 12 months, CAC progression was 20.8% in the vitamin K1 group versus 44% in placebo (RR 0.45, 95% CI 0.18–1.15). Although the trend favoured vitamin K1, statistical significance was not achieved.

The VitaVasK trial tested high-dose oral vitamin K1 (5 mg three times per week) in haemodialysis patients with pre-existing coronary calcification [38]. Although the trial was terminated early because of recruitment difficulties, vitamin K1 supplementation markedly increased serum vitamin K and reduced inactive MGP levels. CT imaging analyses suggested attenuation of thoracic aortic and coronary calcification progression. These findings support a potential anti-calcification signal for intensive K1 supplementation, but the open-label design, limited sample size and early termination require cautious interpretation.

By contrast, vitamin K2 trials in dialysis have been largely neutral for imaging endpoints. Oikonomaki and colleagues randomized haemodialysis patients to MK-7 supplementation and found improvement in vitamin K status but no significant attenuation of aortic calcification progression [39]. The RenaKvit study similarly showed that MK-7 improved vitamin K status, but did not hinder or modify progression of arterial calcification in dialysis patients [40]. In the Trevasc-HDK trial, MK-7 supplementation reduced dp-ucMGP but did not reduce vascular calcification progression, MACE or mortality [41]. MK-7 supplementation may still influence arterial stiffness in selected haemodialysis subgroups, such as patients with diabetes, but these signals require confirmation and should not be equated with proven calcification or outcome benefit [42]. The VIKIPEDIA trial is a multicentre, randomized, open-label, placebo-controlled study evaluating high-dose MK-7 supplementation (1000 µg/day for 18 months) in 44 patients receiving peritoneal dialysis. Its primary endpoint is change in pulse wave velocity, with cardiovascular events and mortality included as secondary endpoints, but definitive outcome data have not yet been reported [43].

Taken together, supplementation with either vitamin K1 or vitamin K2 generally improves biochemical markers of vitamin K status, particularly dp-ucMGP. Across available randomized trials, vitamin K1 has generated the more consistent, although still inconclusive, evidence for slowing imaging-based calcification progression, particularly in selected high-risk cohorts and haemodialysis populations receiving intensive dosing. However, these signals derive largely from small and methodologically heterogeneous studies and should be considered hypothesis-generating rather than clinically actionable. Differences in dose, formulation, route of administration, study population, baseline calcification burden, adherence, vitamin K status, and imaging modality may explain the variability in findings. No trial has demonstrated a reduction in MACE, cardiovascular mortality, or all-cause mortality. Studies reporting clinical events were underpowered and yielded neutral results. Vitamin K supplementation therefore cannot currently be recommended routinely to prevent vascular calcification or cardiovascular events in CKD, consistent with its absence from contemporary cardiovascular guidelines [44] (Fig. 2). Publication bias cannot be excluded, as some studies remain available only as protocols or conference abstracts, while small neutral or prematurely terminated trials may be less likely to reach full publication. Ongoing studies, including VIKIPEDIA, and complete reporting of neutral and negative findings will be important for a balanced assessment of the evidence.

**Figure 2:**
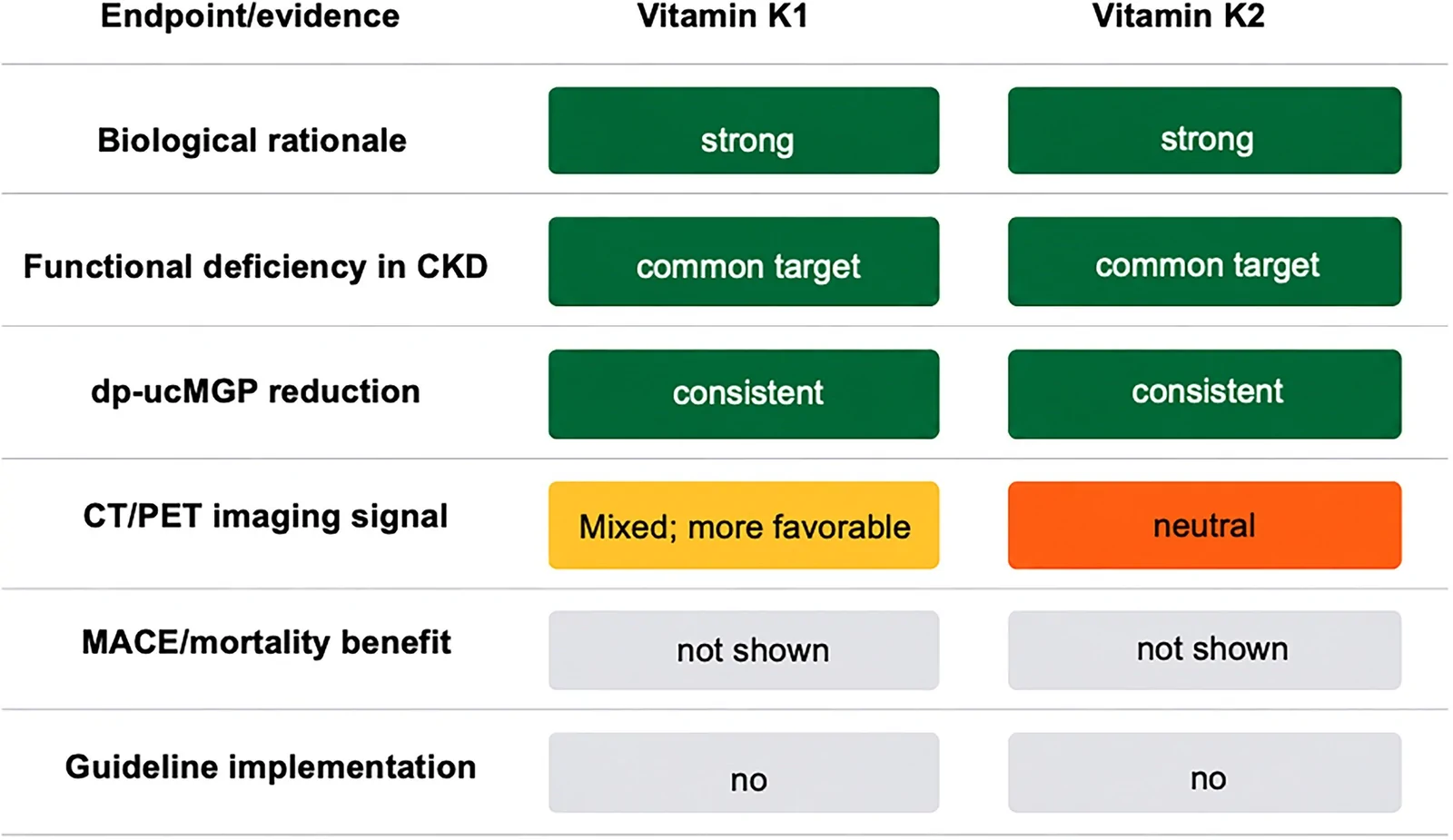
Evidence map of vitamin K supplementation in vascular calcification. The figure summarizes the current evidence for vitamin K1 and vitamin K2, mainly MK-7, across different levels of evidence. Both vitamin K1 and vitamin K2 have a strong biological rationale and consistently improve biochemical markers of vitamin K status, particularly dp-ucMGP. However, effects on CT- or PET-based calcification endpoints remain heterogeneous. No randomized trial has demonstrated a reduction in major adverse cardiovascular events or mortality. Abbreviations: CKD, chronic kidney disease; CT, computed tomography; dp-ucMGP, dephosphorylated-uncarboxylated matrix Gla protein; MACE, major adverse cardiovascular events; MK-7, menaquinone-7; PET, positron emission tomography.

## IMPLICATIONS FOR PRACTICE

Vitamin K1 or K2 should not be prescribed routinely to prevent or treat vascular or valvular calcification, reduce arterial stiffness, or lower cardiovascular risk in CKD or dialysis, because no reproducible clinically meaningful benefit has been established. dp-ucMGP should not be used to initiate or titrate supplementation. Supplementation remains appropriate for established indications, including documented or strongly suspected vitamin K deficiency and the management of vitamin K antagonist-associated over-anticoagulation. In selected high-risk patients, particularly haemodialysis patients not receiving vitamin K antagonists, individualized vitamin K1 supplementation may nevertheless be considered after discussion of the uncertain benefit and favourable safety profile. Enrolment in clinical trials should remain the preferred approach.

## FUTURE OUTLOOK

Future trials should move beyond repeating short studies based on conventional calcification scores (Table 2). Patient selection should be refined by CKD stage, dialysis status, baseline calcification burden, and vitamin K status, with particular focus on patients with marked functional deficiency and active but potentially modifiable calcification. Studies should also distinguish medial, intimal, and valvular calcification, as these phenotypes may differ in mechanism and treatment response. More informative endpoints, including plaque composition, calcification density, and 18F-NaF PET activity, should complement conventional CT calcium scores. Vitamin K form, dose, route, and duration should be standardised. Direct comparison of K1, K2, and placebo with both imaging and clinical endpoints would be more informative than further single-form studies. In haemodialysis, intravenous administration may improve adherence and avoid variable gastrointestinal absorption. Concomitant treatments should also be balanced or adjusted for, as phosphate binders, calcimimetics, statins, antibiotics, and bile acid sequestrants may influence calcification or vitamin K availability [5, 45–49]. Ultimately, trials must determine whether vitamin K reduces cardiovascular events, heart failure, hospitalization, or mortality rather than merely lowering dp-ucMGP. Adequately powered outcome trials would require large sample sizes, long follow-up, and substantial funding. At present, industry interest appears limited, and investigator-initiated trials face practical barriers.

**Table 2:** Lessons for future vitamin K trials.

Limitation of previous trials	Consequence	Future design implication
Heterogeneous populations	Difficult comparison across non-CKD, CKD, transplant and dialysis cohorts	Stratify by kidney function and dialysis status
Variable baseline calcification	Low event signal in some participants	Enrich for established but potentially modifiable calcification
Inconsistent vitamin K deficiency assessment	Potential dilution of treatment effect	Stratify or enrich by dp-ucMGP or other markers
Different forms and doses	No direct inference on K1 versus K2	Direct K1/K2/placebo trials with standardized formulations
Biomarker response used as efficacy signal	dp-ucMGP is not validated as outcome surrogate	Use dp-ucMGP as pharmacodynamic marker only
CT calcium score as dominant endpoint	Cannot distinguish plaque stabilization from harmful calcification	Add plaque composition, density or 18F-NaF PET
Short follow-up	May miss slow biological effects	Longer follow-up and repeated imaging
Underpowered clinical outcomes	No conclusion on MACE or mortality	Larger multicentre outcome-oriented trials

Abbreviations: CKD, chronic kidney disease; CT, computed tomography; dp-ucMGP, dephosphorylated-uncarboxylated matrix Gla protein; K1, vitamin K1/phylloquinone; K2, vitamin K2/menaquinone; MACE, major adverse cardiovascular events; PET, positron emission tomography; RCT, randomized controlled trial.

## CONCLUSION

Vitamin K is biologically plausible as a strategy to inhibit vascular calcification. However, randomised controlled trials have shown mixed effects on the progression of vascular and valvular calcification, with no demonstrated benefit for cardiovascular events or mortality. Although vitamin K1 has shown somewhat more favourable effects on imaging-based calcification progression than vitamin K2, particularly in selected haemodialysis cohorts, these findings remain hypothesis-generating rather than clinically actionable. Current evidence therefore does not support routine supplementation to prevent calcification or improve cardiovascular outcomes.

## Data Availability

No new data were generated or analysed in support of this manuscript.
